# Acetabular roof stress fracture: a rare cause of hip pain in children

**DOI:** 10.11604/pamj.2016.24.288.8855

**Published:** 2016-07-29

**Authors:** Zied Jlalia, Mehdi Bellil, Maher Ben Ghachem

**Affiliations:** 1 Orthopedic Pediatric Department, Kassab Institute, Tunis, Tunisia; 2 Orthopedic Department, Charles Nicole Hospital, Tunis, Tunisia; 3 Orthopedic Pediatric Departement, Children Hospital, Tunis, Tunisia

**Keywords:** Stress fracture, acetabulum, magnetic resonance imaging

## Abstract

Stress fracture of acetabular roof is an unusual cause of hip pain. It is considered as an underdiagnosed entity. People who are more susceptible to experience this fracture are athletes, soldiers and dancers. We present the case of an 11 year old girl with a roof acetabular stress fracture for which the diagnosis and follow-ups were possible by the means of MRI. The treatment was keeping the child at a complete rest. Failure to abide with this treatment can cause the stress fracture to evaluate into a complete fracture.

## Introduction

Stress fractures are injuries that occur as a result of repeated cyclical loading of the bone. These fractures are seen when a bone with normal resistance undergoes pressure and excessive efforts. The diagnosis is not always evident since the first tests usually don't show the injury. It is only in the clinical setting and by examining the activities of the patient that we can suspect the fracture. We present the case of an 11 year old girl suffering from a limp right leg pain with no notion of trauma. Standard radiographs and CT scan of the pelvis were inconclusive and only the evolution of the case and Magnetic resonance imaging (MRI) led to the diagnosis.

## Patient and observation

**Figure 1:**
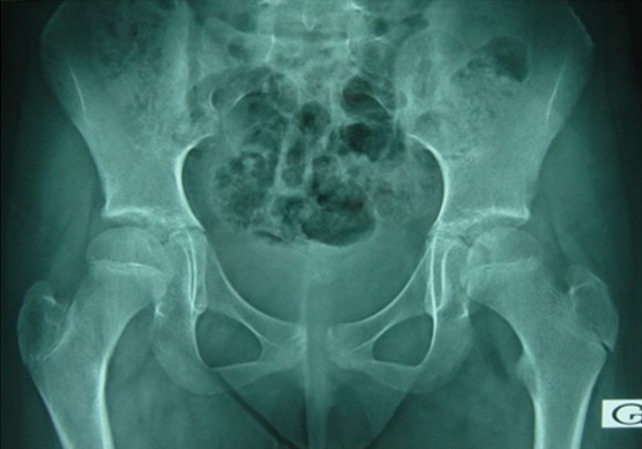
Frontal radiography of the pelvis

**Figure 2:**
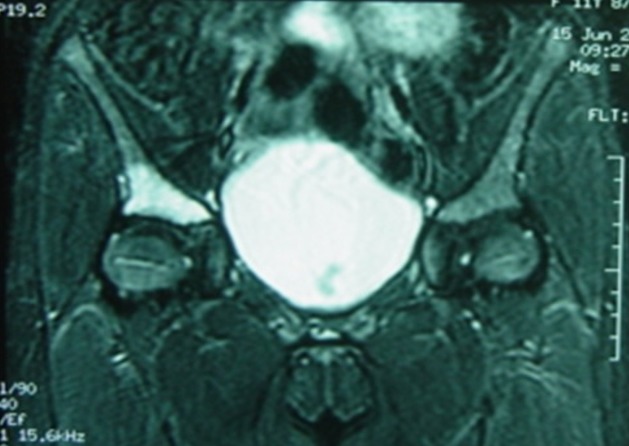
First MRI, hyperintense signal on T2 in acetabular roof

**Figure 3:**
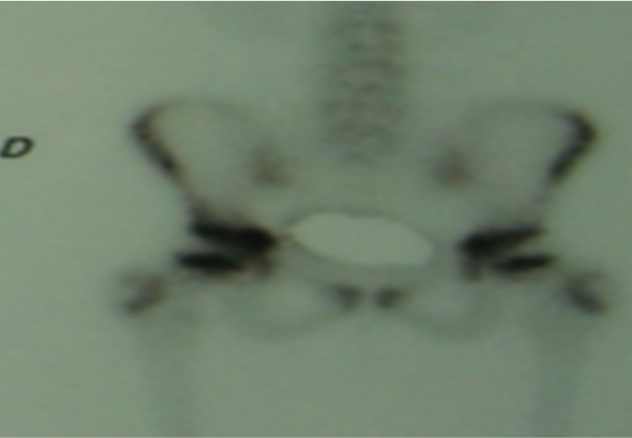
Scintigraphy, increased up take at the right Acetabulum

**Figure 4:**
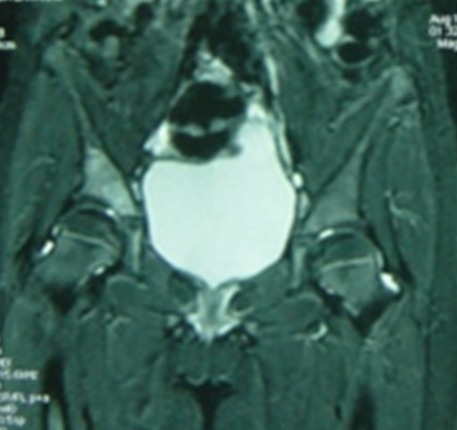
3 months after first MRI, reduction of oedema in the roof of the acetabulum without bone lesion

**Figure 5:**
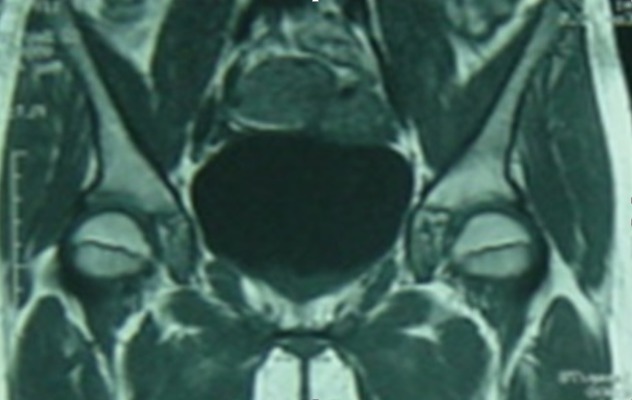
6 months after first MRI: complete regression of the oedema and the high signal on T2

This is the case of an 11 year old girl suffering from right hip pain, examined over the past four months in a context of a pyrexia. The child neither has any significant medical history nor takes any drug treatment. She does not practice sport regularly. The pain is present both during activity and occasionally at rest. It begins at the groin and radiates in the anterior aspect of the thigh. On clinical examination, hips were normal with symmetrical mobility. The monopodial support and jump were painful and unstable. Direct axial impaction of the hip joint elicited no pain. Standard radiographs and CT scan of the pelvis were normal (Figure 1). Magnetic resonance imaging (MRI) was prescribed, it showed isogonal on T1 and hyper-intense signal on T2 sequence of the acetabular roof, without bone destruction or invasion of the hip joint (Figure 2). This abnormal signal can not be associated with a particular pathology. The bone scintigraphy shows increased uptake at the right acetabulum (Figure 3). Laboratory tests especially markers of infection were normal. The duration of the development and the absence of bone destruction makes the tumoral etiology unlikely. Diagnosis of stress fracture of acetabulum roof was retained. Conservative treatment consisting of discharge of the right leg was followed for 3 month, walking with cane one the healthy limb was allowed. After this period, the pain decreased remarkably and MRI showed a diminution of oedema in the roof of acetabulum (Figure 4). After 6 months the child was completely free of pain and the physical exam was perfectly normal. MRI showed a complete regression of the oedema and the signal of the acetabulum was normal corresponding to the healing stage of the stress fracture (Figure 5). Follow up was during a year. During this year, the patient did not complain of any sort of pain. She goes through full recovery activity and sport.

## Discussion

Stress fracture appears on the bone segments, such as lower extremities especially tibia, fibula, pelvic ring and feet, when enduring mechanical stress. Subject to shear stress on the neck femoral, pubis and its branches, in addition to the compression level of the acetabulum and the femoral head region, the hip is a site exposed to stress fracture [1]. Stress acetabular fracture in young patients is extremely rare. It is exclusively experienced by distance runners 21% military recruits 31%, and ballet dancers, with a female predominance [1]. In a study over 2 years in the Ports mouth naval medical center, WILLIAMS and al [2] found 12 cases of acetabular stress fractures in recruits military split into two patterns. Seven patients had acetabular roof stress fractures with two cases of bilateral acetabular roof stress fractures and one synchronous tensile sided femoral neck stress fracture. The remaining five patients had anterior column stress fractures, almost always occurring with inferior pubic ramus stress fracture [3]. Clinical diagnosis is not always easy. Occasionally some of these fractures can be misdiagnosed [4]. According to some doctors, in the case of unclear diagnosis, particularly in clinical context, biopsy of the bone is justified [3]. They also believe that the biopsy can stimulate the consolidation of the stress fracture. Indeed a recent study indicated that nearly 80% of the patients with stress fractures attending a rheumatology department presented with associated osteoporosis [3]. The main symptom of the stress fracture is a sudden onset or rapidly progressive pain preventing sport and mono-podal support [1]. When the fracture affects the hip, there is a limitation of very small amplitudes in the passive motion. The diagnosis of this fracture is not easy. That is why it is important to be aware of this condition. It should be considered among painful lower-extremity syndromes particularly within high risk groups (athletes, dancers, soldiers).

Plain radiographs of the pelvis and hip may show a subtle sclerotic band parallel to the acetabular roof. This band is caused by trabecular compression and callus formation. It may also be completely normal. Radiographs are insensitive to diagnose stress fracture but suffice to exclude sinister lesions such as bone tumors. Further investigations with bone scintigraphy or MRI, which are highly sensitive but less specific, are necessary [3]. According to MILLER and al [5] false negative bone scintigraphy has been reported in rare cases. In the study of NACHTRAB and al [3] MRI showed lesions in muscle and tendon insertions that can accompany muscle stress fracture of hip region. MRI appearances may mimic tumors, but a specific diagnosis of stress fracture can be based on MRI morphology and location of the lesions. The CT scan sensitivity is low concerning stress fractures especially in the pelvis. Bone scintigraphy is highly sensitive for the detection of stress injury in the bone. Its sensitivity approaches 100% and can be positive as early as 72 hours after onset of symptoms [6]. The specificity is lacking but it is very useful in establishing the diagnosis [7]. The stress fracture is usually a benign lesion but some complications can occur, like the extension of a fracture line, and acetabular fracture may be complicated by a protrusion of the femoral head [8]. In most cases the treatment of stress fracture is rest and a period of at least six to eight weeks is usually enough for the complaints to disappear and fracture to unite [9].

## Conclusion

Stress fractures are common over-use injuries seen mostly in athletes and military recruits. These injuries are rare in a pediatric population. They often affect bone segments of the lower limbs particularly the tibia and the fibula, the hip can be also be affected. Pain is the main symptom. The only challenge, as far as these fractures are concerned, is diagnosis especially that plan radiographics do not show any abnormality during the first stages. Further diagnostic studies such as computed tomography, scintigraphy and magnetic resonance imaging (MRI) are necessary. MRI is the best way to diagnose stress fractures. It can also track there progress. Furthermore, awareness of this condition is a key aspect in early diagnosing and efficient treatment before evolution into complete fracture.
